# The Typical Flight Performance of Blowflies: Measuring the Normal Performance Envelope of *Calliphora vicina* Using a Novel Corner-Cube Arena

**DOI:** 10.1371/journal.pone.0007852

**Published:** 2009-11-18

**Authors:** Richard J. Bomphrey, Simon M. Walker, Graham K. Taylor

**Affiliations:** Department of Zoology, University of Oxford, Oxford, United Kingdom; University of Hull, United Kingdom

## Abstract

Despite a wealth of evidence demonstrating extraordinary maximal performance, little is known about the routine flight performance of insects. We present a set of techniques for benchmarking performance characteristics of insects in free flight, demonstrated using a model species, and comment on the significance of the performance observed. Free-flying blowflies (*Calliphora vicina*) were filmed inside a novel mirrored arena comprising a large (1.6 m
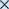
1.6 m
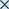
1.6 m) corner-cube reflector using a single high-speed digital video camera (250 or 500 fps). This arrangement permitted accurate reconstruction of the flies' 3-dimensional trajectories without the need for synchronisation hardware, by virtue of the multiple reflections of a subject within the arena. Image sequences were analysed using custom-written automated tracking software, and processed using a self-calibrating bundle adjustment procedure to determine the subject's instantaneous 3-dimensional position. We illustrate our method by using these trajectory data to benchmark the routine flight performance envelope of our flies. Flight speeds were most commonly observed between 1.2 ms^−1^ and 2.3 ms^−1^, with a maximum of 2.5 ms^−1^. Our flies tended to dive faster than they climbed, with a maximum descent rate (−2.4 ms^−1^) almost double the maximum climb rate (1.2 ms^−1^). Modal turn rate was around 240°s^−1^, with maximal rates in excess of 1700°s^−1^. We used the maximal flight performance we observed during normal flight to construct notional physical limits on the blowfly flight envelope, and used the distribution of observations within that notional envelope to postulate behavioural preferences or physiological and anatomical constraints. The flight trajectories we recorded were never steady: rather they were constantly accelerating or decelerating, with maximum tangential accelerations and maximum centripetal accelerations on the order of 3 *g*.

## Introduction

Insects achieve remarkable flight performance, and the literature is replete with observations and measurements to prove this: from the astonishing prey capture rates of darter dragonflies (up to 98%) [1] to the 4500**°s^−1^** angular velocities achieved by courting dolichopodid flies [2]. One can also find many excellent descriptions of specific flight manoeuvres, including such exotica as Immelmann turns in tabanids [3] and pursuit manoeuvres in syrphids [4]. The ability to attain extremes of flight performance may be important in some species, but in most cases, the more limited portion of the flight envelope in which the insect spends the majority of its time is likely to be at least as significant in determining selection pressures upon flight performance. Measurements of the habitual flight performance of insects are surprisingly rare, which is in part due to the absence of any standardized methodology applicable to a wide range of species, and also reflects the difficulty of extracting reliable measurements of performance from noisy biomechanical data. In short, there is currently no answer to the question, “how fast does a fly typically fly?”

Typically, insects are small and fast-moving, which makes it inherently difficult to track their position. Previous studies have tracked insects using a variety of techniques, from simple single camera systems, which assume approximately 2-dimensional motion [4], [5] to sophisticated tracking camera systems [6], [7], onboard transponders for harmonic radar experiments [8], [9] and onboard electromagnetic search coils [10], [11], [12]. Such systems have been used most commonly in the lab, although Dahmen and Zeil [13] developed a method for using synchronised 16 mm film cameras in the field (demonstrated by mapping the trajectories of a lesser housefly, *Fannia cannicularis*, patrolling the airspace beneath a lamp shade). Perhaps the most sophisticated camera system used to investigate insect flight to date is that developed by Fry and colleagues [6], [7], which uses paired pan-tilt cameras to obtain high resolution images of small insects flying in a large volume (approx. 20 m^3^). Each technique has its own merits, from large range [8], [9], to separating the kinematics of head and thorax [10], [11], [12]. The aim of this work is to devise a simple, robust and effective methodology for recording trajectory data under unencumbered flight conditions.

In recent years high-speed digital video camera technology has improved sufficiently to allow footage of free-flying insects to be captured with very high spatial and temporal resolution. Since the resulting data are already digitized, it is also possible to automate analysis procedures, permitting collection of far larger sets of data than has previously been practical, but the fundamental problems of camera synchronisation and calibration remain. We present a simple method for obtaining high quality 3-dimensional data from free-flying insects using a single high-speed camera. This is allied with a rigorous photogrammetric analysis using custom-written software to automate tracking, calibration and measurement procedures. In addition, we deal with the ubiquitous problems of signal processing by using the autocorrelation function of the noise we remove to objectively determine an appropriate filter cut-off frequency. The first part of the paper presents the method and apparatus for the acquisition of insect trajectories. The second part describes a process by which positional data can be transformed into performance envelope parameters and how they, in turn, can be interpreted for comparative analyses. The paper is illustrated by anaylsis of the trajectories and translational flight performance of blowflies (*Calliphora vicina* Robineau-Desvoidy) roaming within our apparatus.

## Materials

### 1. Overview

**Figure 1:**
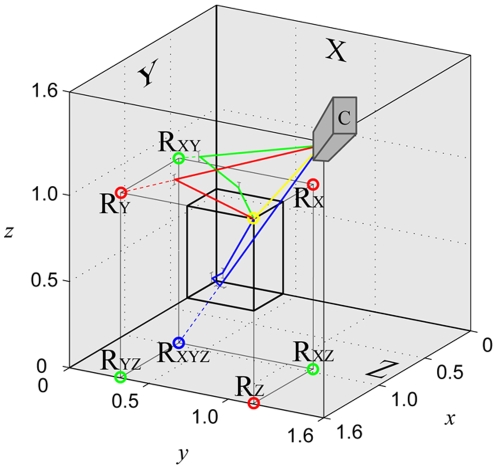
Diagram showing how the primary, secondary, and tertiary reflections of a fly are formed in a corner-cube reflector: yellow circle represents the fly itself; red circles represent the apparent locations of the three primary reflections (ray shown reflecting off the *Y* mirror in this case); green circles represent the apparent locations of the three secondary reflections (ray shown reflecting off the *XY* mirror pair in this case); the blue circle represents the tertiary reflection off all three mirrors (*XYZ*).

Corner-cube reflectors (three plane mirrors joined to form one corner of a cube) are commonly used in long-range optics applications because of their special property of reflecting an incident ray back along a parallel path. Their usage is widespread: from highly technical applications (e.g. Apollo 11's placement of a corner-cube reflector array on the moon to measure its distance from Earth using laser ranging) to consumer products (e.g. bicycle retro-reflectors). Here we exploit a second useful property of corner-cube reflector geometry, which is relevant in close-range applications when an object is placed within the volume of a corner-cube. Any object placed within a corner-cube reflector has seven reflections when viewed from the opposite corner (Fig. 1): three primary reflections (each reflected off one mirror), three secondary reflections (each reflected sequentially off two mirrors) and one tertiary reflection (reflected sequentially off all three mirrors). This property makes the corner-cube reflector an extremely useful tool for photogrammetric applications in which several views of a target are required, although we are not aware of any previous applications in this context. Here we use a large corner-cube reflector together with a single high-speed camera to obtain high-quality photogrammetric measurements of the three-dimensional trajectories of free-flying blowflies (*Calliphora vicina*).

### 2. Animals

Blowflies (*Calliphora vicina* Robineau-Desvoidy) were reared from larvae obtained from a local tackle shop (Fat Phil's Angling Centre, Oxford, U.K.). Adult flies were released into the arena for recording flight performance within a day or two of emergence.

### 3. Flight Arena

**Figure 2:**
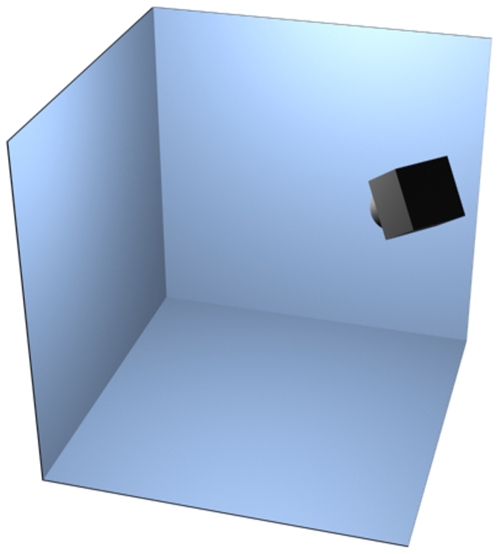
Schematic of the mirrored corner-cube flight arena with camera position and orientation.

A large corner-cube reflector (Fig. 2) was constructed from three 1.6 m square back-silvered mirrors mounted orthogonally on hardwood supports and an aluminum frame (Flexlink Aluminium Structural System, RS Components Ltd. Northhants, U.K.). The dihedral angles between the mirrors were accurate to 90±0.5°, although such precision is not essential because any misalignment is accounted for in the self-calibration procedure described below. The three open faces of the cube were hung with white sheeting and the two sheeted vertical faces were backlit using two cool-running HMI daylight lamps (125 W ARRI Pocket Par, ARRI Group, London, U.K.) to give flicker-free illumination without unduly heating the arena. A single Photron APX monochrome high-speed digital video camera (Photron Europe Ltd., Bucks, U.K.; 1024×1024 pixels at 250 or 500 fps) was mounted at the corner of the cube opposite the reflector corner. The camera was fitted with a Tamron 17–35 mm lens aimed and focused at the reflector corner, giving good depth of field across the working volume. All experiments were conducted under ambient conditions of room temperature and humidity.

The light intensity distribution was approximately gaussian across each sheeted vertical face, giving large-scale contrast to the visual environment and supplementing the finer-scale contrast provided by creases in the sheeting. The sheet forming the roof of the arena was somewhat dimmer than the sides as it could not be lit directly. The most prominent structure of the visual environment was created by the intersections of the mirrors. Because these were back-silvered, each intersection resulted in a gap between the reflecting surfaces of 6 mm. When reflected, these gaps created a shape consisting of three orthogonal intersecting lines of apparent length 3.2 m. A further visual stimulus was provided by the lens of the camera and its reflections. In summary, while no attempt was made to simulate a natural visual environment, the flies were provided with a range of visual stimuli including both horizontal and vertical cues.

### 4. Protocol

Several flies were released into the arena at a time and allowed to fly about freely. Occasionally, the flies had to be stimulated to fly by lightly tapping the glass or sheeting on which they had alighted. The camera was manually post-triggered to capture 27 separate bouts of flight solicited from among 14 flies (the identity of the individual could not be determined, so the results inevitably involve pseudo-replication). These 27 sequences represent all those we collected in which a fly and all 7 of its reflections were visible to the camera.

## Methods

### 1. Automated Tracking

The 27 sequences we collected comprise >5500 frames, each containing eight images of the fly. This corresponds to >44000 coordinates, which were determined automatically using custom-written tracking software in Matlab (Matlab v7.1.0. The Mathworks Inc.). The automated tracking procedure used a tri-layered algorithm which first located the eight images of the fly in a frame, then used image processing tailored to each image in order to determine the position of its centroid, and finally passed that output on to set the search area for the following frame.

In step one, the frame was compared to a reference frame in which no fly was visible. Background subtraction was then used to leave a greyscale image in which only the fly and its reflections were visible. This image was then thresholded at as low a level as possible to leave only eight patches of white against a solid black background, corresponding to the eight images of the fly. These patches include both the wings and body, so do not themselves give a consistent estimate of the fly's position when its wings are flapping. A second step was therefore needed to identify which of the pixels in a patch actually corresponded to the fly's body.

In the second step, the algorithm re-examined the original greyscale values of the eight patches of pixels identified in step one. As the eight images of the fly were not of equal intensity and had varying contrast with the background of the frame (e.g. the tertiary reflection was never as dark as the direct image), each image was analysed separately. Thresholding was applied at a level higher than before and tailored to the local contrast distribution. This had the desired effect of excising the paler regions corresponding to the wings, leaving only the darker pixels corresponding to the fly's body. Canny edge detection was then used to find the outline of each image of the fly's body, and the centroid of each outline was then used to give the pixel coordinates of each image of the fly.

In step three, the pixel coordinates were used to limit the area over which the background subtraction and thresholding of step one were applied to the subsequent frame, by using eight restricted search regions centred on the eight images of the fly in the previous frame. These restricted search regions were tuned to be large enough to take account of the fly's movement from one frame to the next, but small enough to avoid confusing different images of the fly. This adaptive final layer of the algorithm greatly reduced the time taken to analyse each sequence and also reduced errors associated with finding the images of the fly. If one of the images of the fly could not be found in a particular frame (most commonly when an image passed over a mirror edge or met another image), the tracker continued searching the same area in subsequent frames until the image reappeared. The missing pixel coordinates were then linearly interpolated between frames, although in practice this is not critical because there are always enough other images of the insect visible to obtain an accurate measurement of its position later. The end result of this step of the procedure was a set of two-dimensional pixel coordinates for the eight fly images in every frame.

### 2. Photogrammetry

The next stage of the analysis consists in using the two-dimensional pixel coordinates of the eight images of each frame to determine the three-dimensional laboratory coordinates of the fly. The apparent three-dimensional locations of the seven reflections are uniquely determined by the three-dimensional position of the fly and the optical properties of the corner-cube reflector. For example, each primary reflection appears to lie the same perpendicular distance behind the mirror as the fly lies perpendicularly in front of it. This is of course true of any object reflected in a plane mirror, and the same reasoning can therefore be extended to infer the apparent locations of the secondary and tertiary reflections. If the corner-cube reflector is orthogonal then the fly and its seven reflections together form the vertices of a virtual cuboid centred on the corner of the mirrors and oriented with its edges normal to the mirrors (Appendix S1 and Fig. 1). The situation is more complicated if the mirrors are not orthogonal, but the structure of the eight images remains uniquely constrained by the constant geometry of the corner-cube reflector (Appendix S2). This known structure means that the eight images of every frame can effectively be used as a virtual calibration object. However, whereas a conventional calibration object would have known dimensions but unknown position and orientation with respect to any external coordinate system, the virtual calibration object formed by a corner cube reflector has unknown dimensions but known position and orientation with respect to the corner-cube. This structure also allows us to use the relative pixel coordinates of the images to identify whether each is an image of the fly or one of its reflections, and if the latter then to determine the sequence of mirrors in which the image was reflected (Appendix S2).

The constrained structure of the eight images in each frame can be exploited using a common photogrammetric technique known as bundle adjustment to calibrate the system [14]. Bundle adjustment is normally used in multi-camera applications, but is here adapted to the case of a single camera pointing into a corner cube reflector. The great advantage of bundle adjustment techniques is that they use all of the measurements (i.e. each captured frame) to optimize the model parameters and target coordinates simultaneously, resulting in the best possible fit to the data. If the model parameters are estimated from the measurements without reference to a separate calibration (as is the case here), then the bundle adjustment is said to be self-calibrating. Bundle adjustment uses large scale optimization techniques to fit a photogrammetric model in which the estimates of the camera parameters and target coordinates are jointly optimal. Here we also include the geometry of the corner-cube reflector in our functional model to allow us to estimate the dihedral angles of the mirrors and thereby account for any non-orthogonality in their placement.

Appendix S2 describes the photogrammetric model we used, which includes the dihedral angles between the mirrors (dealing with the effects of corner-cube non-orthogonality), principal point offset in the camera (displacement of the principal axis from the centre of the image plane), radial distortion (variation in angular magnification with angle of incidence), tangential distortion (displacement of points in the image caused by misalignment of the lens components) and rectangular pixels (which also has the effect of dealing with shear). The self-calibrating bundle adjustment was performed using nonlinear least squares optimization with explicit outlier removal (Appendix S3).

The standard Levenberg-Marquardt method nonlinear least squares optimization routine used here minimizes the total squared reprojected pixel error for all data points (i.e. the sum of the squared difference between the measured pixel coordinates and those predicted by the estimates of the model parameters and subject coordinates). Individual points with a reprojected pixel error >3.0 pixels were considered outliers and excluded from the model by treating them as missing observations. The mean reprojected pixel error after screening for outliers was <0.8 pixels, which is always less than a quarter of a body length and often very much less, depending upon how close the fly was to the camera. The actual measurement error is of course much better than this because the estimate of the fly's position in each frame is based upon information from all eight images.

Self-calibrating bundle adjustment techniques are able to estimate accurately the geometry of an object or trajectory but are said to be datum deficient in respect of scale, in that the units of the estimated target coordinates are arbitrary. This can only be dealt with by external calibration with respect to some standard measure, and for this purpose we took several images of a 1 m steel rule placed on the floor of the cube. The end result of this step of the procedure was therefore the estimated three-dimensional coordinates of the fly in every frame, along with photogrammetric model parameters and estimates of the measurement error.

### 3. Signal Processing

The aim of this study was to develop a technique which rapidly acquires high quality kinematic data on the translational motion of free-flying insects. We used the photogrammetric method described above to measure the position of blowflies, and then followed the usual approach of determining velocity and acceleration by numerical differencing. This greatly amplifies any error in the position measurements: acceleration, in particular, is acutely sensitive to errors in position, and grossly exaggerated values therefore result if the position data are not filtered to remove noise. Ideally, the position data should be filtered to eliminate as much of the noise as possible without removing any of the underlying signal. This is difficult when the underlying signal is not directly known, but by assuming that any noise is white noise, it is nevertheless possible to determine an appropriate filter cut-off frequency on the basis of the autocorrelation function of the residuals between the filtered and unfiltered data [15]. The reconstructed three-dimensional position data are filtered despite the measurement noise arising from two-dimensional pixel positions. However, because of the different projections, the 2D errors are expected to cancel to some extent and indeed we would expect the assumption of Gaussian noise to be better justified in the three-dimensional estimates than the two-dimensional measurements (e.g. because in the two-dimensional images one pixel movement amounts to a different real-world distance depending on the location within the image field and the projection in question). The choice of filter cut off frequency is not necessarily optimal, rather we have chosen the lowest filter cut-off frequency at which none of the underlying signal was lost, under the assumption of Gaussian white noise.

To determine the cut-off frequency, we first filtered the raw data separately across a range of cut-off frequencies (varying between 1 Hz and the Nyquist frequency in 1 Hz steps). This was done using a zero-phase forward and reverse digital filter using the coefficients of a third-order Butterworth filter. Forward-reverse filtering eliminates phase lag, which is important because the three Cartesian coordinates of position are filtered separately and then combined after differencing to determine the total velocity and acceleration. Forward-reverse filtering also has the effect of doubling the effective order of the filter, thereby producing a sharper frequency response. We next subtracted the filtered data from the raw data at each cut-off frequency, and autocorrelated the residuals to obtain functions normalized by their variance.

The autocorrelation function of a sequence is the average product of the sequence with a time-shifted version of itself [16]. Since white noise is assumed to be completely random, its autocorrelation function is zero at any non-zero time lag (although in practice the autocorrelation function of a finite sample will not be uniformly zero). White noise passed through a linear time invariant processor (e.g. a Butterworth filter) has the same autocorrelation sequence as the filter itself [16]. Hence, if we assume that our raw measurements consist of white noise superimposed upon an underlying autocorrelated signal, then the cut-off frequency for a low-pass filter is the lowest frequency at which the autocorrelation function of the residuals has the characteristics of white noise passed through the same filter. At lower frequencies, the filtering removes some of the autocorrelated signal as well as most of the noise, and the residuals will therefore be autocorrelated in the same way as the portion of the signal which has been removed.

**Figure 3:**
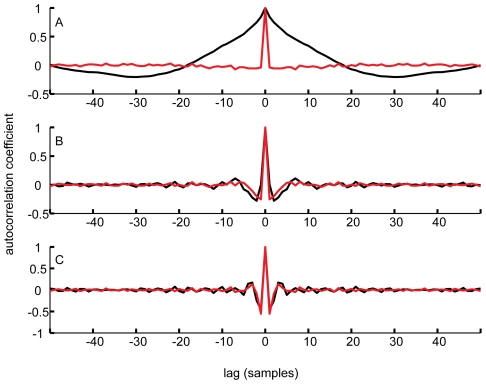
Autocorrelation functions during filtering. Plots of the normalized autocorrelation functions of the residuals of the 500 fps *x*-position data (black lines) after filtering at: (A) 10 Hz, (B) 48 Hz and (C) 100 Hz. For comparison, we also plot the normalized autocorrelation function of the residuals of a sequence of Gaussian white noise of the same length passed through each of the filters (red lines). For this method we selected a cut-off frequency at which the lowest frequency at which the autocorrelation function of the residuals of the actual data still matched closely the autocorrelation function of the residuals of the random sequence - in this case, at around 48 Hz. An animation of the change with respect to cut-off frequency of the autocorrelation (and the variance of the autocorrelation) can be found in Supporting Information.

Fig. 3 plots the normalized autocorrelation function of the residuals of our 500 fps data filtered at three different cut-off frequencies (black line), together with the autocorrelation function of a random sequence of the same length passed through the same filter (red line). With a 10 Hz cut-off (Fig. 3A), the residuals show a high degree of autocorrelation over a wide range of time lags, indicating that the filter has removed some of the signal. At 100 Hz (Fig. 3C), the autocorrelation of the residuals is greatly reduced and the shape of the autocorrelation function is similar to that of the filtered white noise. This remains true down to a cut-off frequency of about 48 Hz (Fig. 3B). We therefore chose a cut-off frequency of 48 Hz as the most conservative (i.e. lowest) filtering frequency which did not remove signal from the unfiltered data. An animation of how the autocorrelation function of the residuals varies with filter cut-off frequency can be found in Supporting Information (Movie S1).

Having determined the appropriate cut-off frequency, we filtered the position data for each sequence separately, appending a short buffer at the beginning and end of each sequence to minimize transient effects caused by the impulse response of the filter. In each case, the data from the first and last 15 points of the sequence were used to extrapolate a further 100 points, which allowed the start-up and stop transients to subside to an insignificant level within the buffer. These buffers were removed from the data immediately post-filtering, leaving a correctly filtered sequence free from unwanted start-up and stop transients. Once filtered, the positional data from each sequence were differenced once to obtain the components of velocity in each of the Cartesian axes, and again to obtain the respective components of acceleration.

Finally, because many flight sequences included a collision with a mirror, collisions were removed from all parts of the data set. Collisions involve extraordinarily high accelerations (sometimes in excess of 8 *g*) and would therefore artificially enlarge the natural flight performance envelope if included in the analysis. We therefore ignored all data points in which either the *x*, *y*, or *z* component of the coordinate was within 14 mm of a mirror (approximately one wing span). A total of 4687 data points remained after collisions and close encounters with the mirrors had been removed. The end result of this final stage of the analysis was a complete set of data describing the translational kinematics of 27 sequences of blowfly flight.

## Results

We begin by describing the statistical distributions of the kinematic variables we measured. The measured data give an indication of the flight performance envelope of blowflies under experimental conditions, although we do not expect to have explored the performance envelope to its limits. Furthermore, because the filter cut-off frequency is chosen not to remove any of the signal, and inevitably leaves in some portion of the noise as a result, we use 99% confidence limits in place of strict maxima or minima when describing the range of routine flight performance. We use a one-tailed confidence limit for unsigned data and two-tailed confidence limits for signed data.

In order to describe the statistical distributions of what are vector quantities, we first decompose velocity and acceleration into scalar components. In the case of velocity, the horizontal direction of flight (i.e. heading) is not relevant to the flight dynamics, and we therefore resolve only the horizontal (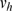) and vertical (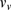) components of total flight speed (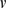). Although 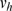 and 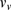 are naturally without sign (because the fly is treated as a particle without a defined forward direction), we adopt the convention of signing 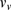 positive if the fly is climbing, and negative if the fly is descending.

In the case of acceleration 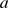, we distinguish between tangential acceleration (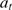, defined as the component of total acceleration tangential to the instantaneous velocity vector) and centripetal acceleration (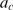, defined as the component of total acceleration normal to the instantaneous velocity vector). Whereas 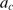 is without sign (because centripetal acceleration is always directed into a turn), we adopt the convention of signing 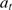 positive if the fly is speeding up and negative if the fly is slowing down. The same convention is used to sign horizontal acceleration, 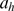, but similar to the convention adopted in respect of vertical speed, we sign vertical acceleration, 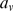, positive or negative according to whether the tangential acceleration vector points up or down, respectively.

**Figure 4:**
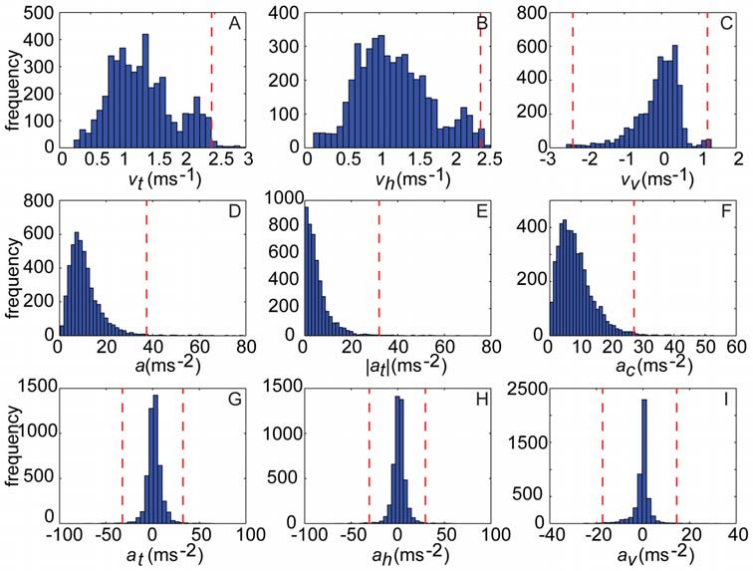
Histograms of translational flight performance. Plots show translational flight performance metrics: (A–C) total speed (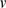) and its horizontal (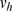) and vertical (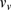) components; (D–F) total acceleration (*a*) and its absolute tangential (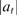) and centripetal (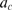) components; (G–I) total tangential acceleration and its horizontal (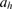) and vertical (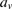) components. In each case, the variables plotted in the first column are the Pythagorean sums of the variables plotted in the second and third columns within a row. Dashed lines represent 99% confidence intervals and are one-tailed in the cases where a single line is presented, and two-tailed where two lines are plotted.

Fig. 4 shows histograms of the various components of velocity and acceleration for all recorded trajectories. Note that the variables plotted in the first column are the Pythagorean sums of the variables plotted in the second and third columns within a row. Fig. 4A–C shows histograms of total (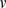), horizontal (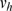) and vertical (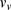) flight speed. Although the data set presented in this study is small, it is perhaps interesting to note at this stage that the distribution of total flight speed appears to be bimodal (Fig. 4A), with modes at approximately 1.2 ms^−1^ and 2.3 ms^−1^. The same apparent bimodality shows in the horizontal (Fig. 4B) and vertical (Fig. 4C) components of total velocity. Further data are required to determine if this is a consistent phenomenon pertaining to a gait transition, from either fast or slow trajectories determined by the individual's motivation, or simply a result of undersampling. In any case, the observed bimodality contrasts with the results of [11], which show a unimodal distribution for both components.

The mean total flight speed was 1.3 ms^−1^ (s.d. = 0.5 ms^−1^), with a maximum of 2.5 ms^−1^. The mean horizontal flight speed was 1.2 ms^−1^ (s.d. = 0.5 ms^−1^), with a maximum of 2.4 ms^−1^. The mean total flight speed we measured in free-flight was a little lower than the 1.65 ms^−1^ mean flight speed found in a tethered flight study by Nachtigall and Roth [17], but much higher than the speeds measured by Schilstra and Van Hateren [11], who used a flight arena with a volume 64 times smaller than ours. Indeed, the maximum horizontal speed attained in their 0.4 m cube (1.2 ms^−1^) was the same as the mean horizontal flight speed in our 1.6 m cube (1.2 ms^−1^). The mean vertical component of velocity of our flies was –0.1 ms^−1^ (s.d. = 0.6 ms^−1^), indicating that on average they descended a little in flight (Fig. 4C). This is not surprising, because the flies were released above the middle of the cube. Of greater physical significance is the observation that the maximum descent rate (–2.4 ms^−1^) was almost double the maximum climb rate (1.2 ms^−1^). This asymmetry is also visible in the strong negative skew of the distribution (skewness = –1.2, defined as the third central moment of 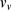 over the cube of its standard deviation), consistent with the directional effect of gravity but counter to the results of Schilstra and Van Hateren [11].

Fig. 4D shows the histogram of the magnitude of total acceleration 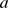. The magnitude of total acceleration is rarely close to zero, indicating that our flies almost never flew steadily. Histograms of the magnitudes of the tangential and centripetal components of total acceleration (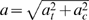) are shown in Fig. 4E–F. These show that while near-zero tangential accelerations are commonplace (indeed, they are the mode), near-zero centripetal accelerations are comparatively rare. This demonstrates that while our flies were capable of maintaining both constant speed and constant flight path direction, they usually only maintained the former. The maximum tangential acceleration (32.0 ms^−2^) was not dissimilar to the maximum centripetal acceleration (27.1 ms^−2^), which indicates that aerodynamic forces of similar magnitude were used in order to turn as to speed up or slow down.

Fig. 4G–I shows histograms of total tangential acceleration 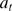 and its horizontal 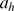 and vertical 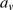 components. The distribution of total tangential acceleration (Fig. 4G) and the distribution of the horizontal component (Fig. 4H) are both approximately symmetric about zero (skewness = –0.1 for both variables). In contrast, the distribution of the vertical component of tangential acceleration is more negatively skewed, presumably reflecting the directional effect of gravity (Fig. 4I; mean = –0.3 ms^−2^; skewness = –0.6).

**Figure 5:**
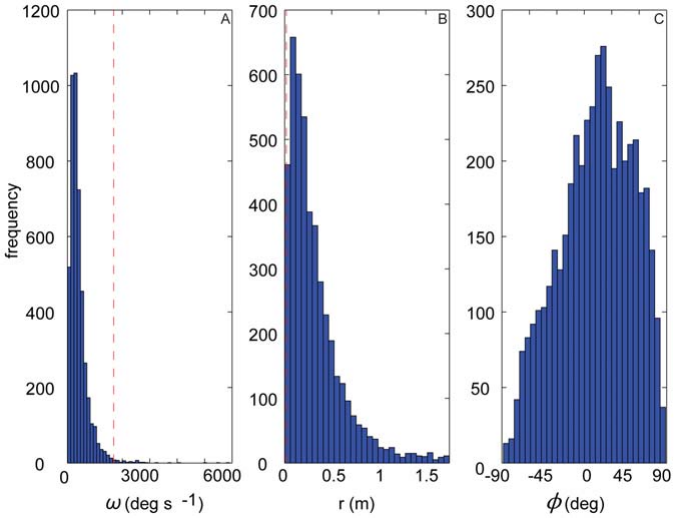
Histograms of turning flight performance. Plots show performance metrics related to turning: (A) turn rate (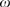); (B) turn radius (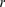) for all 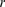
<1.75 m; (C) elevation angle of centripetal acceleration vector (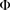). Dashed lines represent one-tailed 99% confidence intervals. No confidence intervals are plotted for 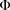 since the data are constrained to ±90°.

Fig. 5 shows histograms of several variables related to turning performance. Fig. 5A plots the distribution of the rate of turn 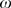, which is a direct measure of agility. We calculated 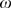 by taking the arcsine of the result of dividing the magnitude of the cross product of successive velocity vectors by the product of their magnitudes. The distribution of rate of turn is unimodal with a strong positive skew (skewness = 4.1). The maximum rate of turn was 1700°s^−1^, with the mode occurring at around 200°s^−1^, showing as above that our flies had a tendency to veer rather than to fly in straight lines.

Fig. 5B plots a histogram of instantaneous turn radius, 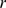, which is a direct measure of manoeuvrability. We calculated 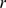 as flight speed (averaged between successive velocity vectors) divided by rate of turn (in rad s^−1^). The right hand tail of the distribution of turn radii is not of interest, because the limit of straight flight corresponds to a turn of infinite radius. Fig. 5B therefore plots only the distribution of turn radii <1.75 m, although as our flies rarely flew in straight lines (i.e. with large-to-infinite turn radius), this subset of data incorporates >98% of the measurements. The minimum turn radius was 0.018 m, although it is clear from the distribution (Fig. 5B) that most turns were accomplished within a much larger radius, with a mode of approximately 0.1—0.2 m (i.e. on the order of 10 body lengths).

Fig. 5C plots the angle which the centripetal acceleration vector makes with the horizontal, 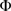. This is zero during horizontal turns and straight flight, but varies continuously during any turn with a vertical component. The range of possible values (–90°
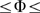
90°) is almost entirely explored. The maximum and minimum possible values are attained when turning away from the horizontal in a pull-down or pull-up manoeuvre: the relative paucity of measured values at ±90° therefore indicates that pull-down and pull-up manoeuvres are usually inclined to one side or the other. The mean angle which the centripetal acceleration vector makes with the horizontal is 13° (s.d. = 40°): this indicates that over most of the time recorded, turns were accomplished as manoeuvres involving a moderate degree of pull-up.

**Figure 6:**
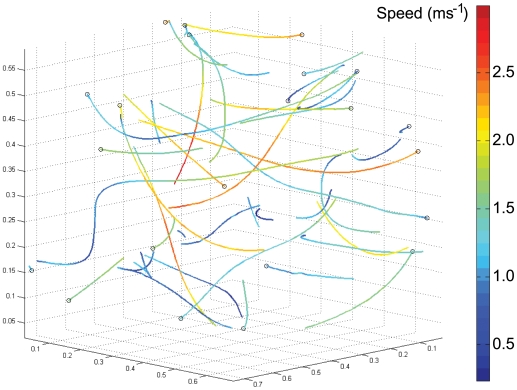
Flight trajectories coloured by speed from low speeds (coloured blue) to high (coloured red). See colour bar for detail. Trajectories closer to any mirror than 14 mm have been removed.

**Figure 7:**
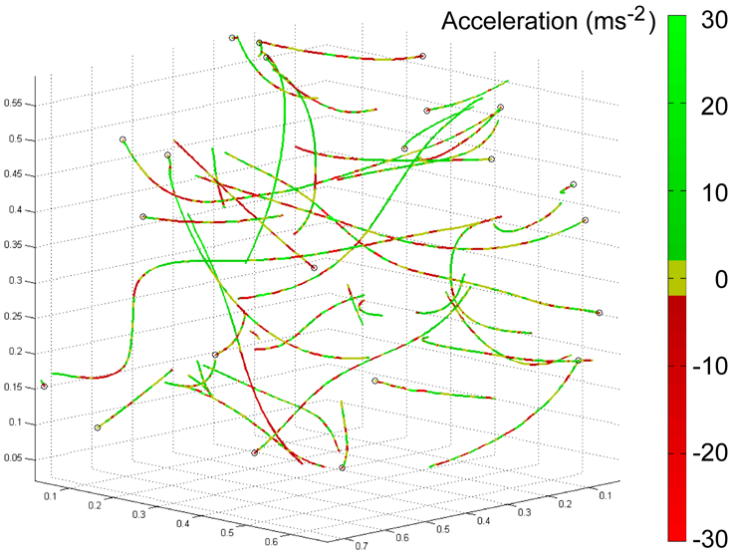
Flight trajectories coloured by tangential acceleration (ms^−2^). Near-zero accelerations are coloured yellow; positive accelerations are coloured green; decelerations are coloured red. See colour bar for details.

Fig. 6 and Fig. 7 plot all of the recorded flight trajectories in three-dimensional space, with line colour as a function of total speed and total tangential acceleration, respectively. The trajectories include a range of manoeuvres with flight paths consistent with the ‘banked turns’, ‘dives’ and ‘zigzags’ described by Schilstra and Van Hateren [11], although we lack the data on body orientation that are strictly necessary to distinguish between these. We saw no obvious examples of ‘U-turns’ [11], perhaps because the flies tend to collide with the mirrors rather than avoiding them, while all of our examples of ‘reverse turns’ [11] seem to occur after collisions. Fig. 6 offers some evidence that the apparent bimodality in total flight speed (Fig. 4A) reflects trajectories being either largely ‘fast’ (coloured orange through red, corresponding to the mode around 2.3 ms^−1^) or largely ‘slow’ (coloured blue through green, corresponding to the mode around 1.2 ms^−1^). In other words, individual trajectories tend to involve one or other of the modal flight speeds, but not both. Again, this may be due to motivational, gait, or sampling issues. Fig. 7 shows that every one of the 27 trajectories involves periods of both speeding up (coloured green) and slowing down (coloured red), frequently interchanging between the two modes repeatedly. Thus, whilst flight speed is held more or less steady for short periods (Fig. 6 and Fig. 7), it is unusual for flight speed to be held steady over an entire trajectory.

## Discussion

### 1. Performance Envelope

The various components of flight performance described in the previous section are not expected to be maximised simultaneously. For example, at the maximum achievable flight speed, the tangential acceleration must, by definition, be zero. Thus, the flight performance envelope of any flying vehicle or animal represents a series of trade-offs which in themselves offer insight into the functioning of the system. These may be subdivided into physical constraints (e.g. maximum net thrust must vary with flight speed because of the effects of drag), physiological constraints (e.g. the flight motor may only be physiologically able to operate maximally at a particular speed) and behavioural preferences (e.g. the fly may prefer not to fly as fast as it is able). Hence, if we can correctly identify the physical constraints within which the system must operate, we may be able to gain insight into the physiological constraints and behavioural preferences that have evolved.

In examining such constraints, it is crucial to work with variables which are mathematically independent. Velocity and acceleration are independent, and each has three degrees of freedom. However, because the horizontal direction of flight is irrelevant to the flight dynamics, we may lump the 2 horizontal components of velocity together and consider only the flight speed (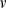) and flight path elevation (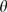). In the case of acceleration, we resolve the acceleration into its net components tangential and normal to the flight path, specifying the elevation of the normal acceleration by its angle to the horizontal (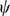) and noting that the elevation of the tangential acceleration is already specified by 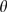. The tangential and normal components of acceleration can be thought of as aerodynamic forces normalized by body mass, and are in fact identical to the ‘relative thrust-drag’ (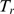) and ‘relative lift’ (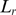) defined by [18] after [19].

**Figure 8:**
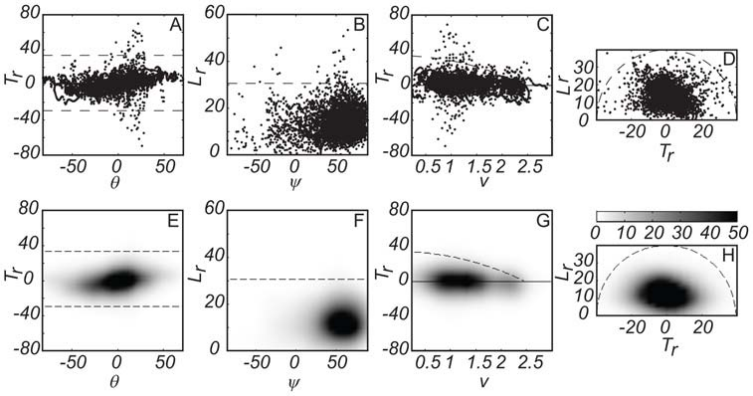
Flight performance envelopes. Scatter plots (A–D) and density plots (E–H) of flight performance data: (A,E) relative thrust-drag (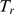) against flight path elevation angle (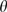); (B,F) Relative lift (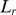) against its elevation angle (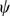); (C,G) relative thrust-drag against flight speed (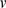); (D,H) relative lift (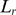) against relative thrust-drag (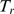). In each case, notional physical limits of the flight performance envelope are plotted on the figures as dashed lines (see body text for detail on limit line construction).

We therefore have a set of five independent variables: flight speed (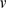), flight path elevation (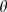), relative thrust-drag (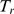), relative lift (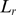), and lift elevation angle (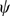). The relationships between these 5 variables have 4 degrees of freedom among them, and we plot the 4 most relevant relationships in Fig. 8. The first row of this figure contains scatter plots of the measured data, while the second row contains density plots of the same data. Figs. 8A,E and B,F plot relative thrust-drag (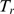) and relative lift (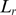) against their respective elevation angles 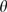 and 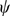, and the 99% confidence limits of 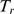 and 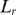 are plotted as dashed lines to mark the approximate physical limits of the flight performance envelope based on our experimental data. Again, note that we do not expect to have captured peak blow fly flight performance in this data set because the flies were not stimulated in a way which would necessarily induce maximal performace, yet the methodology remains valid for comparative analysis since the dashed lines represent the hard limits of our measurements. This is useful since the distribution of points within and relative to these limits reveals behavioural or alternative mechanical constraints. Figs. 8C,G plot relative thrust-drag (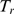) against flight speed (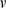). Here, a notional upper physical limit (dashed line) is constructed by assuming that the maximum available thrust is constant (in practice this may not be the case) and by assuming that drag increases quadratically with speed from zero at 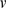 = 0. The equation of the line is determined by taking the upper 99% confidence limit of 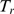 as the maximum of the quadratic, and fitting the line to pass through the value of 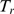 recorded at the 99% confidence limit on flight speed. We make no attempt to construct a lower limit, because the physical constraints upon such a limit are not obvious. Finally, Figs. 8D,H plot relative lift (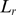) against relative thrust-drag (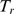), and use the 99% confidence limit of the total self-generated acceleration to suggest the notional physical limit (dashed line).

Because the notional physical limits plotted in Fig. 8 are constructed using the 99% confidence limits of the measured data, it is inevitable that some of the data fall outside, while many of the individual points in the scatter plots overlie one another. The density plots therefore provide a much better visual indication of the distribution of the data. None of the notional physical limits we construct fit the distribution of the data especially closely, and it is clear that in general the preferred flight performance envelope is much narrower than even these sub-maximal flight performance envelopes.

Figs. 8A,E shows that relative thrust-drag and flight path elevation are positively correlated (Pearson's linear correlation coefficient 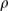 = 0.30; 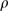 = 0.00001), although the significance of this relationship derives, of course, from the high degree of autocorrelation within a given flight trajectory. In any case, large positive values of relative thrust-drag (i.e. peak thrust) are not used during dives (i.e. large negative flight path elevation), and nor are large negative values of relative thrust-drag (i.e. peak drag or braking) used during climbs (i.e. large positive flight path elevation), as can be seen in the absence of points in the upper left and lower right corners of the rectangular limits. This implies that our flies used the largest tangential thrust-drag forces only when opposing gravity, and did not generate large tangential thrust-drag forces when they did not need to.

The distribution of the measured data in Figs. 8B,F displays a strong asymmetry with respect to the limits on possible performance, with the data points concentrated mostly at high positive lift elevation angles and a mode at around 60°. This distributional asymmetry is not in itself surprising, since the relative lift vector must be elevated in order to counteract gravity. However, the fact that the modal lift elevation angle is <90° is consistent with the earlier observation that our flies tended to veer, turning using an aerodynamic force that is normal to the flight path (i.e. a lift force) and inclined to the vertical (i.e. 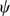
<90°), almost certainly as a result of banking.

The distribution of the measured data in Figs. 8C,G, show that the notional quadratic upper limit on relative thrust-drag seems to do a reasonably good job of predicting maximum flight speed. In other words, the speed at which the limit intercepts the *x*-axis is close to the maximum speed attained, which need not be the case given the method by which the limit is constructed.

In terms of scatter, the semi-circular notional physical limit on relative lift and thrust-drag encompasses the measured data reasonably well, consistent with the interpretation that there is a maximum available aerodynamic force which may be directed normal or tangent to the flight path according to the orientation of the fly [11]. As expected, the modal relative lift is close to 1 *g*, and the result of this is that the cloud of data points is displaced a little above the *x*-axis.

### 2. Benchmarking the Blowfly Flight Envelope

Flight trajectories were varied in shape, and distributed throughout the flight volume. The wider range of flight speeds we measured compared to those in the free-flight experiments of Schilstra and Van Hateren [11] suggest that our results may come closer to exploring fully the natural blowfly flight performance envelope although almost certainly fails to elicit maximal response in this experimental paradigm. The larger performance envelope is probably partly because our flies were not encumbered by trailing wires and search coils (an advantage of the photogrammetric method), and partly because our flight volume was 64 times larger (which had the disadvantage of preventing us from measuring body orientation in this study). In fact, the apparent volume of our flight arena was eight times larger still, taking reflections of the physical arena into account. Evidence that the flies perceived the arena as the larger virtual volume consists in their regular collisions with the mirrors, which were clearly not identified as solid surfaces by the flies' visual systems. Collision episodes were removed from this analysis because they are not relevant to the flight performance envelope but are of separate biomechanical interest.

The performance envelope section makes use of the maximal/minimal values we recorded to construct notional upper (and, where appropriate, lower) physical limits on blowfly flight performance. These limits, and the distribution of the data points within them, are our benchmarks of normal blowfly flight performance. Together they show that our flies only rarely pushed themselves towards the possible limits of flight performance, and instead spent most of their time operating within a fairly narrow and well-defined comfort zone (denoted by the dark patches in the density plots of Figs. 8E–H) leaving plenty of room for manoeuvre. It will be interesting in due course to compare the room for manoeuvre in blowflies with that in other species. For example cruising predators such as hawker dragonflies (Aeshnidae) might be expected to leave a large room for manoeuvre about their typical cruise performance, while capture-dart predators such as darter dragonflies (Libellulidae) might be expected to perform closer to their limits whilst in flight. There will still inevitably be significant evolutionary selective pressure on routine flight performance since the energetic costs of foraging and exploration are likely to be just as important as peak performance. More generally, the benchmarking method we describe here should be suitable for comparative studies of performance across a range of both aerial and aquatic animals.

The distribution of the measured data within the notional maximal flight performance envelope gives an indication of behavioural preference and physiological or anatomical constraint. For example, the fact that our flies did not generate large positive thrust during dives (Figs. 8A,E) may indicate a behavioural preference to reduce power output when possible: if gravity can do the work of acceleration, then let it. An alternative physiological explanation of the same phenomenon is that the musculo-skeletal stresses encountered when pulling out of a very fast dive may be intolerable, so that peak thrusts are never executed in those instances. Similarly, the observation that our flies rarely produced downward-directed lift (Figs. 8B,F) almost certainly reflects the fact that this is difficult, if not impossible, to achieve anatomically without inverting the body or taking negative loads on the wings. Both possibilities are likely to be undesirable for a fly: an inverted posture because of the problems associated with compensatory head roll and a negatively loaded wing because of buckling [20]. Comparative benchmarking studies may shed more light on which are the real preferences and constraints and how these vary between species.

## Supporting Information

Appendix S1 Properties of an ideal corner-cube camera.
(1.17 MB RTF)

Appendix S2 Properties of a real corner-cube camera
(0.86 MB RTF)

Appendix S3 Self-calibration of a corner-cube camera
(0.06 MB RTF)

Movie S1 To show the change in variation of the residuals plotted against filter cut-off frequency. (H264 compression)
(0.95 MB MOV)
