# Supplementary material for: The Typical Flight Performance of Blowflies: Measuring the Normal Performance Envelope of Calliphora vicina Using a Novel Corner-Cube Arena
Source: PLoS One. 2009 Nov 18;4(11):e7852. doi: 10.1371/journal.pone.0007852 (PMC2773008; doi:10.1371/journal.pone.0007852)
Supplement: Appendix S1 — Properties of an ideal corner-cube camera. (1.17 MB RTF) [file pone.0007852.s001.rtf]

Supporting Appendix S1: Properties of an ideal corner-cube camera
 
We begin by constructing a theoretical model of an ideal corner-cube camera, defined as a single camera directed at the vertex of a corner-cube reflector. This will serve as a vehicle for developing understanding, and will provide starting values for the self-calibrating bundle adjustment described in Supporting Appendix S3. The ideal corner-cube camera is assumed to consist of a single distortion-free camera positioned so that its principal axis is collinear with the intermediate axis of three orthogonal planar mirrors at which it points (Fig. 2). These assumptions are relaxed during the self-calibration procedure described in Supporting Appendix S2 to account for the effects of lens distortion, principal axis offset and mirror non-orthogonality.
We first model the optical properties of the ideal corner-cube reflector at which the camera points. Let the intersections of the three mirror planes define the axes of a Cartesian frame, so that any point in three-dimensional space may be represented by the vector . Reflections of  will be seen by the camera in each of the three mirrors. These primary reflections are described by the matrices:

		

in which the subscript denotes the axis normal to the plane of the mirror in which the reflection is seen. Applying these matrices to  gives the apparent locations of the three primary reflections:
 
		

Each primary reflection is reflected again by each of the other two mirrors. These secondary reflections are described by the matrices:

		
Applying these matrices to  gives the apparent locations of the three secondary reflections: 

		

Note that if the mirrors are exactly orthogonal, then the apparent location of the secondary reflections is independent of the sequence of reflection. Hence, because the same location can only appear to fall behind one mirror, it follows that only one of the two possible sequences of secondary reflection will be visible from any given viewpoint.
Each secondary reflection is reflected again by the third mirror. This tertiary reflection is described by the matrix:

		

(in which the sequence of multiplication is arbitrary provided that the mirrors are orthogonal). Applying this matrix to  gives the apparent location of the tertiary reflection: 

		

which is again independent of the sequence of reflection if the mirrors are exactly orthogonal. It is informative to note, by comparing Eqs. ,  and , that the locations of a target point  and its reflections together form the eight vertices of a cuboid at .
We now model the formation of an image of a point  and its seven reflections in a distortion-free camera. Let the two-dimensional projection of  onto the camera's image plane be denoted . The origin  of the image coordinate system is assumed to coincide with the principal point , defined as the point at which the camera's principal axis intersects the image plane. This ideal case is described by the well-known central perspective projection: 

		

where the variable  can take any positive real value, reflecting the fact that one camera image of a point is sufficient to determine the line on which the point falls but not sufficient to determine its distance from the camera. The coordinates  denote the position of the perspective centre  in the Cartesian frame defined by the mirrors. The principal distance  measures the distance between the perspective centre  and the principal point . The  matrix is a rotation matrix accounting for the effects of camera orientation: its nine elements are functions of only three independent parameters and there are therefore a total of seven independent parameters in the central perspective projection model.
The third equation of Eq.  may be used to eliminate  from the first two equations to write the so-called collinearity equations: 

		
 
		

Equivalent equations may be written down for each of the seven reflections of  by changing the signs of ,  and  according to the axes in which reflection occurs. Assuming that the  coordinates of the images of a point  and its seven reflections are known, we have a total of sixteen equations in three unknown variables and seven parameters.
