# Supplementary material for: The Typical Flight Performance of Blowflies: Measuring the Normal Performance Envelope of Calliphora vicina Using a Novel Corner-Cube Arena
Source: PLoS One. 2009 Nov 18;4(11):e7852. doi: 10.1371/journal.pone.0007852 (PMC2773008; doi:10.1371/journal.pone.0007852)
Supplement: Appendix S2 — Properties of a real corner-cube camera (0.86 MB RTF) [file pone.0007852.s002.rtf]

Supporting Appendix S2: Properties of a real corner-cube camera

  We now relax several of the assumptions made in our ideal model to account for the effects of lens distortion and mirror non-orthogonality. We will begin by modelling the optical properties of a corner-cube reflector in which the mirrors are not exactly orthogonal. Let one of the mirrors (call this ) be taken as a reference plane whose normal defines the direction of the -axis in a Cartesian frame. Now let a second mirror  be added approximately orthogonal to , and let the intersection of  and  define the direction of the -axis, thereby fixing the Cartesian frame. Finally, let a third mirror  be added approximately orthogonal to  and .
Since the bottom mirror  has been taken as a reference plane, its reflection matrix  must be identical with the matrix  defined in Eq. (1):

		(10)

The mirror  contains the -axis, so its reflection matrix  is obtained by applying a rotation about the -axis (call this ) to the matrix  defined in Eq. (1):

		(11)

The reflection matrix  for  is obtained by applying a rotation about the -axis (call this ) followed by a rotation about the rotated -axis (call this ) to the matrix  defined in Eq. (1):
 
		(12)

As before, the secondary and tertiary reflection matrices are obtained by multiplying the respective primary reflection matrices, but it is easy to show that this operation no longer commutes as it did before. Hence, the apparent locations of the secondary and tertiary reflections depend now on the sequence of reflection, which can be identified from the segment in which a reflection falls. The intersections of the mirrors divide the image into three  segments, such that the  segment in which a secondary reflection falls identifies the mirror in which it was last reflected. In the same way, the reflections of the mirror intersections further subdivide the image into six  segments, such that the  segment occupied by the tertiary reflection identifies in which of the six possible sequences it was reflected. Hence, once the sequence of secondary and tertiary reflection is identified, it is possible to rewrite the right-hand sides of the sixteen collinearity equations with the addition of only three more parameters (,  and ) to deal with the effects of non-orthogonality in the mirrors.
We now deal with the effects of lens distortion. This is a standard problem in photogrammetry, and a typical model would allow for principal point offset (displacement of the principal axis from the centre of the image plane), radial lens distortion (variation in angular magnification with angle of incidence) and tangential lens distortion (displacement of points in the image caused by misalignment of the lens components). All of these effects can be dealt with by modifying the left-hand sides of the collinearity equations (Eqs. 8-9), which may be rewritten as:


(13)


(14)

where . Here,  and  are the coordinates of the principal point in the image frame; ,  and  are coefficients of a sixth order polynomial representing radial distortion; and  and  are coefficients of a second order polynomial representing tangential distortion. Furthermore, as  are likely to be pixel coordinates in most applications, the possible effects of rectangular pixels are dealt with by the inclusion in Eq. (13) of the pixel aspect ratio, A, defined as pixel width over pixel height. It is therefore possible to rewrite the left-hand sides of the sixteen collinearity equations to deal comprehensively with the effects of camera distortion using only eight additional parameters. Our final photogrammetric model of a corner-cube camera therefore includes three variables (, , ) and eighteen parameters, the values of which remain to be determined.
