# Supplementary material for: The Typical Flight Performance of Blowflies: Measuring the Normal Performance Envelope of Calliphora vicina Using a Novel Corner-Cube Arena
Source: PLoS One. 2009 Nov 18;4(11):e7852. doi: 10.1371/journal.pone.0007852 (PMC2773008; doi:10.1371/journal.pone.0007852)
Supplement: Appendix S3 — Self-calibration of a corner-cube camera (0.06 MB RTF) [file pone.0007852.s003.rtf]

Supporting Appendix S3: Self-calibration of a corner-cube camera

  We deal now with the problem of corner-cube camera calibration, following the standard photogrammetric practice of using a so-called self-calibrating bundle adjustment to produce jointly optimal estimates of the model parameters and target coordinates. The estimates are jointly optimal in the sense that estimates of the model parameters and target coordinates are adjusted simultaneously using nonlinear least squares optimization to minimize the squared reprojected pixel error. As a classical approach in bundle adjustment, nonlinear least squares is reasonably robust when combined with explicit outlier screening, and has the advantage of being straightforward both to implement and to understand. In summary, the final bundle adjustment consists in minimizing the summed squared difference of the left and right hand sides of Eqs. (13) and (14) (and their equivalents for the various reflections) for all target points remaining after screening for outliers.
In order to enhance the efficiency and robustness of the final bundle adjustment, preliminary estimates of the three-dimensional target coordinates were obtained by parameterizing the central perspective projection model from first principles (Eqs. 8 and 9) and their equivalents for the various reflections) and then using nonlinear least squares optimization to solve for the target coordinates frame by frame. For the tracking problem considered here, the estimated target coordinates from each frame were used to provide starting values for the next frame's optimization. Convergence was, as a result, very rapid. The principal distance  was approximated as the nominal focal length of the lens, while the rotation matrix and coordinates of the perspective centre  were determined by assuming that the camera pointed directly at the intersection of the three mirrors from the opposite corner of the cube. The end result of this step of the procedure was therefore a set of preliminary estimates of the target coordinates in each frame.
These preliminary estimates of the target coordinates were then used as starting values in an intermediate bundle adjustment minimizing the summed squared difference of the left and right hand sides of Eqs. (13) and (14) (and their equivalents for the various reflections) for all of the target points together. This intermediate bundle adjustment was used to screen for outliers prior to the final bundle adjustment. All parameters of the camera model were free to vary from starting values set to be equivalent to the central perspective projection used to obtain the preliminary estimates of the target coordinates. The residuals were then used to identify outliers as those image points or reflections with a reprojected pixel error  pixels. This threshold was chosen by examining histograms of the residuals and looking for discontinuities in the error distribution. In every case, these outliers corresponded to secondary or tertiary reflections for which the sequence of reflection had been incorrectly identified. The end result of this step in the procedure was to provide near-final estimates of the model parameters and target coordinates and to screen for outliers.
The parameter and coordinate estimates from the intermediate bundle adjustment were then used as starting values in the final bundle adjustment, which was done on the data after zero-weighting the outliers. Since the distribution of the reprojected pixel error was approximately normal and since the maximum reprojected pixel error was already less than the outlier threshold, it was not deemed necessary to screen the data again. The end result of this step in the procedure was therefore to provide final estimates of the model parameters and target coordinates. The numerical values of the latter are determined to within an unknown scale factor, as can easily be seen by inspection of Eqs. (13) and (14), whose right hand sides are clearly unaffected numerically by multiplying all three-dimensional coordinates by a constant.
The collinearity equations for a corner-cube camera are therefore datum deficient in respect of an unknown scale factor, which we calibrated by placing a steel rule of known length (1m) on the bottom mirror so as to span most of the field of view. We then used the estimates of the model parameters from the final bundle adjustment to solve for the three-dimensional coordinates of the ruler's corners using nonlinear least squares optimization, and solved directly for the undetermined scale factor using Pythagoras' theorem to determine the length of the ruler in the dimensionless coordinate system. We then multiplied all of the three-dimensional coordinate estimates from the final bundle adjustment by this scale factor to give dimensional estimates of the target coordinates that are jointly optimal with the model parameters. With this step, the self-calibration procedure is complete.
